# Effects of a Lifestyle Intervention in Routine Care on Short- and Long-Term Maternal Weight Retention and Breastfeeding Behavior—12 Months Follow-up of the Cluster-Randomized GeliS Trial

**DOI:** 10.3390/jcm8060876

**Published:** 2019-06-19

**Authors:** Julia Hoffmann, Julia Günther, Lynne Stecher, Monika Spies, Dorothy Meyer, Julia Kunath, Roxana Raab, Kathrin Rauh, Hans Hauner

**Affiliations:** 1 Else Kröner-Fresenius-Centre for Nutritional Medicine, Klinikum rechts der Isar, Technical University of Munich, Georg-Brauchle-Ring 62, 80992 Munich, Bavaria, Germany; julia.hoffmann@tum.de (J.H.); julia.guenther@tum.de (J.G.); lynne.stecher@tum.de (L.S.); monika.spies@tum.de (M.S.); dora.meyer@tum.de (D.M.); julia.kunath@tum.de (J.K.); roxana.raab@tum.de (R.R.); kathrin.rauh@tum.de (K.R.); 2 Competence Centre for Nutrition (KErn), Am Gereuth 4, 85354 Freising, Bavaria, Germany

**Keywords:** weight retention, breastfeeding, lifestyle intervention, pregnancy, obesity prevention, routine care, gestational weight gain, postpartum, long-term, follow-up

## Abstract

Postpartum weight retention (PPWR) is associated with an increased risk for maternal obesity and is discussed to be influenced by breastfeeding. The objective was to evaluate the effect of a lifestyle intervention delivered three times during pregnancy and once in the postpartum period on PPWR and on maternal breastfeeding behavior. In total, 1998 participants of the cluster-randomized “healthy living in pregnancy” (GeliS) trial were followed up until the 12th month postpartum (T2_pp_). Data were collected using maternity records and questionnaires. Data on breastfeeding behavior were collected at T2_pp_. At T2_pp_, mean PPWR was lower in women receiving counseling (IV) compared to the control group (C) (−0.2 ± 4.8 kg vs. 0.6 ± 5.2 kg), but there was no significant evidence of between-group differences (adjusted *p* = 0.123). In the IV, women lost more weight from delivery until T2_pp_ compared to the C (adjusted *p* = 0.008) and showed a slightly higher rate of exclusive breastfeeding (IV: 87.4%; C: 84.4%; adjusted *p* < 0.001). In conclusion, we found evidence for slight improvements of maternal postpartum weight characteristics and the rate of exclusive breastfeeding in women receiving a lifestyle intervention embedded in routine care, although the clinical meaning of these findings is unclear.

## 1. Introduction

Obesity prevalence is increasing worldwide and represents one of the major public health concerns [1,2,3]. In particular, increasing obesity rates are observed in women of childbearing age [4,5]. Moreover, a high pre-pregnancy body mass index (BMI) as well as excessive weight gain during pregnancy are strong contributors to the overall risk of obesity later in life [6,7,8]. Accordingly, excessive gestational weight gain (GWG) is discussed to be an independent risk factor for both short- and long-term postpartum weight retention (PPWR) [9,10,11,12]. Breastfeeding may decrease PPWR, and the duration of breastfeeding has been found to be inversely associated with the woman’s risk of overweight [13,14,15]. Despite this evidence, women with obesity are more likely to fail in breastfeeding and are more susceptible to long-term PPWR and inter-pregnancy weight gain, which contributes to a vicious cycle of obesity progression [16,17]. 

Ultimately, both the prevention of PPWR and the initiation of breastfeeding seem to be key determinants in the prevention of obesity in the postpartum (pp) period. Therefore, various randomized controlled trials (RCTs) offering antenatal and pp lifestyle interventions were conducted to tackle excessive GWG and persistent PPWR as well as to promote breastfeeding. Meta-analyses confirm that antenatal lifestyle interventions are effective in limiting GWG and in reducing long-term maternal PPWR until the 12th month pp [18,19]. However, the RCTs included small to moderate numbers of participants, and interventions were usually conducted in community settings and academic facilities [13,20,21,22,23]. Approaches embedded in the real-life setting of routine antenatal care are urgently needed to not only assess their effect on excessive GWG but also on long-term PPWR in order to realistically estimate their potential to interfere with the cycle of obesity progression.

The large-scale, cluster-randomized “Gesund leben in der Schwangerschaft” (“healthy living in pregnancy”, GeliS) trial was designed as a public health approach embedded in the German antenatal routine care system. The primary aim of the GeliS study was to reduce the proportion of women with excessive GWG according to the weight gain recommendations of the Institute of Medicine (IOM) [24] by offering a comprehensive lifestyle intervention alongside antenatal routine visits [25]. Although the intervention was not effective in limiting excessive GWG [26], it yielded some improvements in dietary (manuscript under revision) and physical activity behavior (manuscript under revision) [27,28]. The long-term influence on maternal health remains to be investigated. Herein, our aim was to investigate the effect of the GeliS intervention on short- and long-term maternal PPWR and on the women’s breastfeeding behavior assessed in a 12 month follow-up. Moreover, we sought to identify factors influencing long-term PPWR and maternal breastfeeding behavior.

## 2. Materials and Methods

### 2.1. The GeliS Study: Design and Setting

As a prospective, multicenter, cluster-randomized, controlled, open intervention trial, the GeliS study was performed alongside the German routine health care system in five administrative regions in Bavaria. Pairwise cluster-randomization was conducted by randomly matching two districts (clusters) per region according to birth figures and sociodemographic and geographic criteria [25]. This randomization resulted in one control district (C) and one intervention district (IV) per region. Within each of these districts, the study was performed in gynecological and midwifery practices and thus under real-life conditions in the German routine antenatal care system. Further details about study design, setting, and randomization process were described previously [25].

The study was conducted according to current local regulatory requirements and laws as well as in accordance with the declaration of Helsinki. The study protocol was approved by the Ethics Commission of the Technical University of Munich (project number 5653/13) and was retrospectively registered at the ClinicalTrials.gov Protocol Registration System (NCT01958307) [29].

### 2.2. Participants and Lifestyle Intervention

Participants were recruited between 2013 and 2015 in 71 participating gynecological and midwifery practices (*n* = 39 in IV and *n* = 32 in C). Eligible were women with (1) a pre-pregnancy BMI between ≥ 18.5 kg/m^2^ and ≤ 40.0 kg/m^2^, (2) a singleton pregnancy, (3) age between 18 and 43 years, (4) sufficient German language skills and (5) stage of pregnancy before the end of the 12th week of gestation. Before participating in the study, all women gave their written informed consent for inclusion. As described previously, miscarriage or late loss of pregnancy, terminations, pregnancy complications that interfered with the intervention, and maternal death during the course of the trial were considered as exclusion criteria [25,26]. During the follow-up, women were defined as drop-outs if they were no longer reachable, did not provide contact details, or withdrew participation. Participants in the C attended standard antenatal care and obtained only limited information on a healthy antenatal lifestyle and the importance of breastfeeding by means of a flyer. The IV received a comprehensive lifestyle intervention program.

The lifestyle intervention program was already described in detail elsewhere [25]. Alongside routine care visits, the IV received three antenatal (12th–16th, 16th–20th, and 30th–34th week of gestation) and one pp (6th–8th week pp) face-to-face counseling sessions lasting between 30 and 45 min, which were given by previously trained midwives, medical personnel, or gynecologists. In these lifestyle sessions, women in the IV were informed about adequate GWG according to the recommendations of the IOM and were encouraged to monitor their weight gain weekly using a weight gain chart [24]. Moreover, healthy dietary and physical activity behavior during pregnancy and the pp period were addressed in accordance with national and international recommendations [30,31]. The importance of breastfeeding for both mothers and their offspring was outlined in both the third antenatal counseling (30th–34th week of gestation) and the pp session (6th–8th week pp). In accordance with German breastfeeding recommendations [32], participants were encouraged to exclusively breastfeed their infants until at least the end of the 4th month after delivery. Furthermore, they were informed about potential barriers to initiate breastfeeding and strategies to overcome these. Additionally, infant hunger and satiety signals were discussed. In case of contraindications of breastfeeding, counselors were advised to refer participants to experts.

Subsequent to the lifestyle intervention, women of both groups underwent the same follow-up observation program with data collection until the 12th month pp. The last participant completed the 12 months follow-up at the end of 2017.

### 2.3. Data Collection and Outcomes

Baseline characteristics were collected using a screening questionnaire at the time of recruitment (before the 12th week of gestation, visit 0 (V0)). Weight was measured in participating gynecological or midwifery practices during the course of pregnancy and 6–8 weeks pp (time point 1 postpartum (T1_pp_)) and was retrieved from maternity records. Weight at the 12th month pp (time point 2 postpartum (T2_pp_)) was self-reported on a questionnaire included in a set of questionnaires (QT2_pp_, completed at T2_pp_). If QT2_pp_ was missing, weight was recorded during a phone interview, which was scheduled at T2_pp_ with the main purpose of obtaining data on infant anthropometrics. In QT2_pp_, women additionally indicated whether they were pregnant at T2_pp_.

Pre-pregnancy BMI was calculated based on self-reported pre-pregnancy weight. GWG was defined as the difference between the last measured weight before delivery and the first measured weight at the time of recruitment (V0). Excessive GWG was categorized according to the thresholds provided by the IOM [24]. Short-term PPWR was defined as the difference between the measured weight at 6–8 weeks pp (T1_pp_) and the first measured weight at V0. Long-term PPWR was calculated as the difference between self-reported weight at the 12th month pp (T2_pp_) and the first measured weight at V0. For any calculations based on V0 weight, self-reported pre-pregnancy weight was used if the measured V0 weight was missing, which was the case for 27 participants. A PPWR of 5 kg was shown to be associated with later obesity [33,34,35,36]. Therefore, the threshold of >5 kg was chosen to define clinically relevant PPWR at T1_pp_ and T2_pp_, respectively.

At T2_pp_, data on maternal breastfeeding behavior were collected within QT2_pp_ via questions adapted from the German wide survey “German Health Interview and Examination Survey for Children and Adolescents” (KiGGS) [37]. In QT2_pp_, women were asked to indicate whether they breastfed their offspring at any time (defined as any breastfeeding) and to estimate the duration of any breastfeeding in months. They additionally stated whether they exclusively breastfed, meaning breastfeeding without offering any formula or complementary food to their infant, and were asked to estimate the duration of exclusive breastfeeding in months. Women were defined as meeting the German breastfeeding recommendations if they reported exclusive breastfeeding until the end of the 4th month pp [32].

Data on early antenatal well-being were collected before the 12th week of gestation using the German version of the 5-item World Health Organization Well-Being Index (WHO-5). This is a questionnaire consisting of 5 questions related to the subjective psychological well-being of respondents [38] and has shown to be a valid and reliable indicator of well-being [38,39]. The WHO-5 has been applied across a wide range of study fields, including the field of obstetrics and gynecology, and has been used in comparable clinical trials [38,40]. An overall score of less than 50% has been found to indicate poor well-being and has been used to screen for the diagnosis of depression [38,39]. Analyses using the WHO-5 index were performed as described by Topp et al. [38]. Women with a WHO-5 score lower than 50% were defined as having low antenatal well-being.

Symptoms of postpartum depression (PPD) were assessed at T1_pp_ using the validated German version of the Edinburgh Postnatal Depression Scale (EPDS) [41]. Having an EPDS score of 13 or above was defined as showing symptoms of PPD. Herein, we define PPD as having met the score threshold of the EPDS.

### 2.4. Statistical Analysis

Power calculation was conducted based on the primary study outcome (excessive GWG) and was described elsewhere [25]. All analyses presented herein were performed using SPSS software (IBM SPSS Statistics for Windows, version 24.0, IBM Corp, Armonk, NY, USA).

Analyses for T1_pp_ included all participants except for those who dropped out during the active intervention phase (from inclusion until T1_pp_) due to miscarriage or late loss of pregnancy, terminations, pregnancy complications that interfered with the intervention, maternal deaths, or for other reasons (e.g., change of residence or decline of further study participation). Data at T2_pp_ were reported for all participants without those who were lost to follow-up from T1_pp_ until T2_pp_. PPWR analyses at T2_pp_ additionally excluded all participants that were pregnant.

As defined a priori [26], associations between GWG or excessive GWG and follow-up outcomes were performed as complete-case analyses and included all participants with available GWG except for those who had preterm delivery (<37th week of gestation) and those who were lost to follow-up.

Baseline characteristics, pp weight characteristics, and breastfeeding information are presented as mean ± standard deviation (SD) or as proportions if appropriate. Due to the cluster-randomized design, linear regression models and binary logistic regression models fit with generalized estimating equations (GEE) were applied to compare differences between IV and C according to Donner et al. [42]. Group differences are presented as estimated mean difference or odds ratio (OR) with 95% confidence interval (CI). Adjusted models were controlled for pre-pregnancy BMI category, pre-pregnancy age, and parity. Time variables (gestational age at inclusion, gestational week at birth, T1_pp_ or T2_pp_) were controlled as confounders for the corresponding outcome variables. We included exclusive breastfeeding as a further covariate in post-hoc analyses for models related to PPWR. Sensitivity analyses were performed to explore if the inclusion of participants with self-reported pre-pregnancy weight influenced findings on PPWR.

To identify predictors of maternal long-term PPWR and factors influencing exclusive breastfeeding and any breastfeeding, IV and C were pooled to form one cohort. For all cohort analyses, unadjusted linear and binary logistic regression models were applied, as well as models adjusted for pre-pregnancy BMI category, pre-pregnancy age, parity, group assignment, and T2_pp_. For the outcome long-term PPWR, gestational age at inclusion was included as a further confounding factor.

For all analyses, *p*-values below 0.05 were considered as statistically significant.

## 3. Results

### 3.1. Participant Flow and Baseline Characteristics

**Figure 1:**
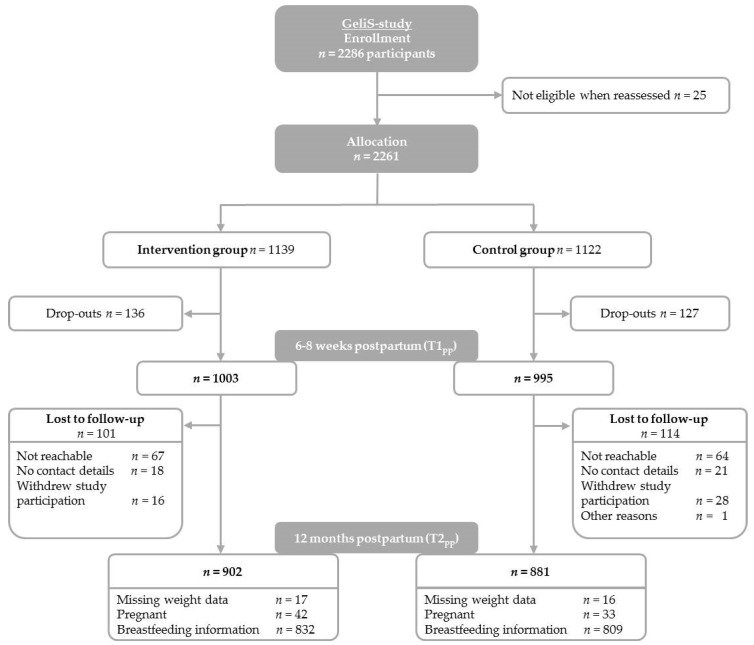
Participant flow.

In total, 2286 women were enrolled in the GeliS trial (Figure 1). Among them, 25 were not eligible after re-assessment of inclusion criteria, resulting in a total of 2261 participants assigned to the IV (*n* = 1139) or the C (*n* = 1122). During the active intervention phase (from recruitment until T1_pp_), 263 participants were lost due to miscarriage or late loss of pregnancy (IV: *n* = 32; C: *n* = 41), terminations (IV: *n* = 3; C: *n* = 6), pregnancy complications that interfered with the intervention (IV: *n* = 4; C: *n* = 0), or several other reasons (IV: *n* = 97; C: *n* = 80) such as change of practice/residence, decline of further study participation, and being no longer reachable. Accordingly, 1998 participants (IV: *n* = 1003; C: *n* = 995) remained in the study until T1_pp_. During the course of the follow-up, 101 participants in the IV and 114 in the C were lost to follow-up, as they were not reachable anymore (IV: *n* = 67; C: *n* = 64), did not provide contact details (IV: *n* = 18; C: *n* = 21), withdrew from study participation (IV: *n* = 16; C: *n* = 28), or due to other reasons (C: *n* = 1), which finally resulted in 1783 participants (IV: *n* = 902; C: *n* = 881) who provided data for follow-up analyses. The drop-out rate between T1_pp_ and T2_pp_ was 10.8%. In total, 21.1% of participants were lost since group allocation.

Baseline characteristics of participants that entered the follow-up are shown in Table 1. Pre-pregnancy weight and BMI were comparable between groups (IV: 68.3 kg and 24.4 kg/m^2^; C: 68.1 kg and 24.3 kg/m^2^). In total, 64.9% of participants showed a BMI between 18.5 and 24.9 kg/m^2^ (normal weight), 23.1% showed a BMI between 25.0 and 29.9 kg/m^2^ (overweight), and 12.0% showed a BMI between 30.0 and 40.0 kg/m^2^ (obesity). IV and C were comparable in terms of pre-pregnancy age, GWG, and educational level. As in the active study phase [26], the IV group included more women that were primiparous (61.8%) in the pp period compared to the C (53.2%).

The proportion of participants lost to follow-up was comparable between groups (IV: 10.1%; C: 11.5%). Participants who were lost to follow-up differed slightly from women entering the follow-up in terms of pre-pregnancy age, parity, educational level, country of birth, history of gestational diabetes mellitus (GDM), and smoking status during late pregnancy (see Table S1).

### 3.2. Weight Outcomes 6–8 Weeks and 12 Months pp

All weight outcomes for the pp period are depicted in Table 2. Women in the IV showed a slightly higher weight at inclusion than in the C (IV: 69.8 ± 13.1 kg; C: 68.9 ± 13.9 kg). Mean PPWR at 6–8 weeks pp was 4.0 ± 4.8 kg in women in the IV and 4.3 ± 4.8 kg in women in the C. At T1_pp_, 38.2% of women receiving lifestyle counseling and 40.9% in the standard care group showed a short-term PPWR above 5 kg (adjusted *p* = 0.643). At T1_pp_, there was significant evidence of a difference between groups in women having the same or a lower weight than before pregnancy (IV: 11.3% vs. C: 14.6%; adjusted *p* = 0.037).

Mean weight loss since delivery until T2_pp_ was significantly higher in women receiving lifestyle counseling compared to the standard care group (IV: 14.3 ± 5.9 kg; C: 13.4 ± 6.0 kg; adjusted effect size 0.85 kg, 95% CI 0.22 to 1.49; *p* = 0.008). The mean long-term PPWR was −0.2 ± 4.8 kg for the IV and 0.6 ± 5.2 kg for the C. In both unadjusted and adjusted models, there was no significant evidence of between-group differences in long-term PPWR related to the first measured weight at inclusion. At T2_pp_, 11.4% of women in the IV and 14.8% in the C showed a PPWR above 5 kg (adjusted *p* = 0.142). Mean PPWR assessed in a per-protocol analysis (data not shown) and the proportion of women at or below the mean pre-pregnancy weight (adjusted *p* = 0.823) did not differ between groups. At the 12th month pp, 40.5% of women in the IV and 40.1% in the C showed a weight at or below pre-pregnancy weight. No difference in PPWR at T1_pp_ and T2_pp_ was found when including participants with the self-reported pre-pregnancy weight if the first measured weight was missing (Table S2).

### 3.3. Breastfeeding Behavior

Table 3 depicts characteristics of breastfeeding behavior. Any breastfeeding during the pp period was reported by 84.7% of women in the IV and 85.2% in the C (adjusted *p* = 0.954). Among breastfeeding women, the proportion of exclusive breastfeeding differed significantly between groups in unadjusted and adjusted models (IV: 87.4%; C: 84.4%; adjusted OR 1.49, 95% CI 1.22 to 1.81; *p* < 0.001). Women in the IV breastfed for 6.6 ± 3.3 months on average and breastfed exclusively for 4.8 ± 1.8 months on average, whereas women in the C breastfed for 6.4 ± 3.2 months on average and breastfed exclusively for 4.7 ± 1.7 months on average. There was no significant evidence of between-group differences in breastfeeding duration, the proportion of women who breastfed longer than 12 months, or the proportion of women who met breastfeeding recommendations (Table 3).

### 3.4. Factors Influencing Long-Term PPWR and Maternal Breastfeeding Behavior

In the GeliS cohort, we found significant evidence for a positive association of PPWR at T2_pp_ with pre-pregnancy BMI category (Table 4; adjusted *p* = 0.012). Age categories were shown to significantly inversely influence PPWR at T2_pp_ (Table 4; adjusted *p* = 0.013) as did educational level (adjusted *p* = 0.001), with women of the highest educational level showing the lowest PPWR compared to women with the lowest educational level (adjusted *p* = 0.001). There was significant evidence of an association between long-term PPWR and excessive GWG (adjusted *p* < 0.001), parity (adjusted *p* = 0.007) and the binary outcome of primiparity before study entry (adjusted *p* = 0.002), and relevant PPWR at T1_pp_ (adjusted *p* < 0.001) and exclusive breastfeeding (adjusted *p* = 0.005), but not between PPWR at T2_pp_ and any breastfeeding, antenatal well-being, or PPD (Table 4). Apart from exclusive breastfeeding (adjusted *p* = 0.012), no interaction with group assignment was found.

Table 5 depicts factors that influenced whether a woman chose to exclusively breastfeed, and Table S3 reports variables that influenced if a woman provided any breastfeeding. Pre-pregnancy BMI category was significantly associated with both any breastfeeding (Table S3; adjusted *p* = 0.002) and exclusive breastfeeding (Table 5; adjusted *p* = 0.001), showing that women with a BMI between 25.0 and 29.9 kg/m^2^ (adjusted *p* = 0.006) or a BMI between 30.0 and 40.0 kg/m^2^ (adjusted *p* = 0.002) were less likely to breastfeed exclusively compared to women with normal weight (BMI between 18.5 and 24.9 kg/m^2^). There was significant evidence for an association between educational level and any breastfeeding (Table S3; adjusted *p* < 0.001) and exclusive breastfeeding (Table 5; adjusted *p* = 0.024), with women with a higher educational level being more likely to adopt the respective breastfeeding behavior. Both antenatal well-being and PPD significantly decreased the odds of any breastfeeding (Table S3; adjusted p value for antenatal well-being *p* = 0.018 and PPD *p* < 0.001, respectively) but not of exclusive breastfeeding. There was no evidence of excessive GWG influencing either of the breastfeeding outcomes (Table 5; Table S3).

## 4. Discussion

To our knowledge, this was the first large-scale trial to evaluate the effect of a lifestyle intervention conducted alongside routine care on short- and long-term PPWR and maternal breastfeeding behavior. The GeliS intervention did not improve maternal pp weight development until delivery [26] but yielded some minor improvements in maternal pp weight development and in the proportion of women who exclusively breastfed compared to standard care, although the clinical meaning of these findings is unclear. While the intervention did not seem to influence short-term PPWR and weight loss from delivery until 6–8 weeks pp, mean PPWR at the 12th month pp tended to be lower in the intervention group. Accordingly, weight loss since delivery until the 12th month pp was found to be more pronounced in women who previously obtained counseling. Although a higher proportion of women reached pre-pregnancy weight 6–8 weeks after birth in the control group, this group difference disappeared at the 12th month pp weight measurement. Findings on the influence of pre- and postnatal lifestyle interventions on maternal PPWR are often inconclusive and depend on the definition of PPWR, participant characteristics, and follow-up period, which complicates a comparison of study outcomes. While some studies reported a positive influence of lifestyle interventions on PPWR [20,43], others found no effect [44,45,46]. A recent meta-analysis suggested a favorable effect of the intervention with an overall weighted mean difference of −0.73 kg (95% CI −1.32 to −0.14; *p* = 0.015) between groups and a weighted mean difference of −0.68 kg (95% CI −1.28 to −0.09; *p* = 0.023) in PPWR 12 months after birth [19]. Although we reported a comparable effect size for long-term PPWR, we found no significant evidence of a between-group difference. This might be explained by the specific study setting of the GeliS trial and the general problem of scaling-up interventions [47]. Unlike GeliS, none of the in the meta-analysis included trials were performed under real-life conditions in the setting of routine antenatal care.

Currently, there is a debate about factors that influence maternal PPWR in the short and the long term and thus might predict the risk for sustained PPWR [48]. We found strong evidence for an association between excessive GWG and long-term PPWR, which was consistently observed by others, confirming excessive GWG as a major risk factor for PPWR even beyond the first year after birth [9,10,24,44,46,49]. Moreover, our findings suggest that parity is associated with PPWR, with multiparous women showing a lower PPWR at the 12th month pp compared to primiparous women. Albeit our observations are consistent with reports of others [48,50], the overall opinion on the role of parity in PPWR is inconclusive [51]. Similar to others, we reported pre-pregnancy BMI category and educational level to be consistent predictors of long-term PPWR [20,52] and found short-term PPWR > 5 kg to be strongly associated with PPWR at one year after birth, but not antenatal well-being and PPD. Furthermore, the only breastfeeding pattern we found to beneficially influence PPWR was exclusive breastfeeding, with no observed effect in mothers who practiced any breastfeeding. Some studies confirm a link between breastfeeding and PPWR [13,14,46], while others fail to provide sufficient evidence [20]. This discrepancy might be explained by the heterogeneity observed in methodological procedures and breastfeeding outcomes. Drawing evidence from our findings and relevant literature, the overall effect of breastfeeding on PPWR seems to depend on breastfeeding intensity and duration. We acknowledge that other determinants such as postnatal diet and physical activity behavior may have a fundamental influence on PPWR, and analyzing their contribution on maternal pp weight development might generate ideas for initiatives to prevent long-term PPWR and inter-pregnancy weight gain [53].

Evaluating the success of lifestyle interventions on a woman’s risk for sustained weight retention, Sagedal et al. [45] demonstrated a broader spectrum of pp weight characteristics beyond PPWR, addressing variables such as returning to pre-pregnancy weight and weight loss since delivery. Our results showed that weight loss since delivery until the first year after delivery was 14.3 kg in the IV and 13.4 kg in the C on average and corresponded to findings by Sagedal et al. (IV: 13.3 kg; C: 14.0 kg) [45] and Phelan et al. (IV: 13.6 kg; C: 12.5 kg) [20], who included participants from all BMI classes. However, only the GeliS trial showed that women in the IV achieved greater weight loss pp, suggesting the effectiveness of our lifestyle intervention for this outcome. Sagedal et al. reported significant between-group differences in women returning to pre-pregnancy weight (IV: 53.2%; C: 43.1%) [45], which is in contrast to results of the “Fit for Delivery” [20] and the GeliS study. Although studies describe effects of lifestyle interventions on pp weight development, its clinical relevance remains to be questioned. PPWRs above 4.55 kg or 5 kg are commonly used as markers to define relevant or sustained PPWR and consequently to assess the clinical relevance of PPWR, as weight retention above these thresholds is associated with obesity later in life [24,33,34,35,36,54]. Our data did not provide any evidence for an effect of the GeliS intervention on relevant PPWR. Unfortunately, relevant PPWR is only reported in a few other trials [21,43,44,46,55]. Among them, only one study found that lifestyle interventions significantly impacted clinically relevant PPWR at the 12th month after birth [44].

Besides improving maternal weight outcomes in the pp period, the GeliS intervention sought to support participants in the initiation of breastfeeding and showed significant between-group differences in the proportion of women who exclusively breastfed (IV: 87.4% vs. C: 84.4%; adjusted *p* < 0.001). However, this difference was rather small, and therefore the clinical meaning of this finding remains unclear. No other breastfeeding outcome appeared to be affected by counseling, which suggests that our intervention was not effective in changing maternal breastfeeding behavior and therefore does not support adoption of our counseling methods in clinical practice. Unfortunately, a comparison with other studies in this field is limited, as few trials included breastfeeding advice or reported relevant data. In a pilot trial that we conducted prior to the GeliS study, we were not able to show between-group differences in the duration of either any breastfeeding or exclusive breastfeeding [44]. Vinter et al. reported similar high numbers of women with obesity initiating breastfeeding in their Danish cohort but no effect of the intervention on breastfeeding outcomes [13]. In the “Fit for Delivery” study, which included women from all BMI classes and did not target breastfeeding behavior, breastfeeding rates were low and did not differ between groups (IV: 10.4% and C: 8.3% at the 6th month pp and 3.4% and 4.6%, respectively, at the 12th month pp). Overall, our breastfeeding rates correspond to other German observations that report an increasing trend towards the initiation of any breastfeeding over the last decade [37,56]. Similar to our observations, German and US studies found educational level and age before or at birth to be predictive for any breastfeeding [37,57]. Moreover, US observational studies extend our findings, showing that BMI category not only influences breastfeeding initiation but also breastfeeding maintenance beyond both the first and the 6th month pp [57]. We found that antenatal well-being and PPD were associated with any breastfeeding but not with exclusive breastfeeding. Others have observed that women with depressive symptoms exclusively breastfed less frequently [58]. Current research expands our findings, reporting inverse associations between antenatal depressive symptoms and the initiation of breastfeeding as well as observations that both antenatal depression and PPD predict early cessation of breastfeeding [57,59,60]. Drawing from evidence presented herein, we suggest future initiatives to offer tailored and personalized counseling according to the individual needs of the mother. This will help to elucidate whether antenatal interventions are effective in achieving improvements in maternal breastfeeding behavior. 

The analyses presented herein have some limitations. Weight was partly self-reported, which is generally thought to provide valid estimates and is widely used in comparable trials [61,62], but remains subjective. We are aware that weight measurements may not be completely standardized, as data were collected in all participating practices by varying personnel. Moreover, we observed inconsistencies in the first measured weight between groups that we attributed to the timing of the first weight measurement and to between-group differences in participants that were lost to follow-up. To address this limitation, we included “time of the first weight measurement” as a covariate in our analyses on PPWR. Power calculation was conducted based on the primary study endpoint (GWG). The power to detect differences in the secondary outcomes, including PPWR and breastfeeding, was not considered in the study design. We found differences between baseline characteristics among women included in follow-up analyses and those who dropped out in the pp period. Although we adjusted for some of these parameters in our analyses (BMI category and parity), this might have introduced bias and could have influenced results. Counseling was not based on concepts of behavioral change theory, which we acknowledge as a shortcoming related to both the training of counselors and the content of the counseling sessions. Our cohort differed slightly from the average German women of childbearing age in terms of educational level and BMI classes and was therefore not completely representative of the general German population [5]. In order to analyze the effect of GWG on PPWR, we combined women with underweight (*n* = 38) and normal weight to one group. Moreover, we pooled women with inadequate and adequate GWG to one group defined as women with non-excessive GWG. We acknowledge that pooling might have biased the overall influence of the dichotomized outcome excessive GWG on PPWR. We are aware that the contribution of other relevant predictors of PPWR, such as smoking, diet, and physical activity, are not included in our analyses. Moreover, we acknowledge that data on breastfeeding behavior were collected retrospectively by a questionnaire, which may limit its validity.

Notwithstanding our limitations, there are several strengths that merit particular attention. Current research in this field is mainly conducted under controlled conditions in community and academic settings or includes only small to moderate numbers of participants [19]. The GeliS trial was designed as a large-scale lifestyle intervention that was implemented under real-life conditions. Assessing whether a public health intervention is successful and ultimately effective largely depends on its scalability. Through our study, we were able to demonstrate that we could implement a lifestyle intervention in the setting of routine care reaching a wider population of our target group. Findings from this study on pp weight development are promising first steps towards reducing obesity risk of women in the pp period on a broader scale. Herein, we reported minor effects on long-term pp weight development and slight improvements in the rate of exclusive breastfeeding. We were able to assess PPWR in women from all BMI categories and to selectively describe the overall influence of BMI on PPWR and breastfeeding behavior on the cohort level. During the follow-up period, we lost only 10.8% of participants and in total—since group allocation until the 12th month pp—only 21.1%. This was a rather low drop-out rate for the pp period considering that other studies reported drop-out rates after birth between 15% and 25% [13,20,45]. We attribute the pp drop-out rate of 10.8% to our effort of thoroughly informing participants about the importance of the long-term follow-up at study entry. Finally, we reported data on a 12 months follow-up, which was longer than in most other trials and contributed valuable information towards estimating the long-term effect of interventions on PPWR. By following participants until the 5th year after birth, we will be able to provide further data on maternal weight development.

## 5. Conclusions

The evidence outlined above demonstrated that the GeliS lifestyle intervention, which was implemented under real-life conditions, yielded some small effects on pp weight development and the rate of exclusive breastfeeding. Moreover, our analysis clearly showed that a considerable proportion of women in both groups retained more than 5 kg weight at the 12th month pp, which increases their risk of obesity later in life. Additional research will elucidate the effect of the GeliS intervention on PPWR beyond the first year until the 5th year after birth and will be able to evaluate the intervention effect on maternal pp weight development and infant outcomes. Effective strategies embedded in routine care that improve maternal breastfeeding behavior and achieve clinically relevant pp weight outcomes are critical for improving both pre- and postnatal health on a large scale. Tailored interventions that consider different high risk subgroups may increase the success of interventions on PPWR and breastfeeding behavior.

## Figures and Tables

**Table 1:** Baseline characteristics of participants entering the follow-up.

	Intervention (*n* = 1003)	Control (*n* = 995)	Total (*n* = 1998)
**Maternal characteristics**			
Pre-pregnancy age, years ^a^	30.2 ± 4.3	30.5 ± 4.6	30.3 ± 4.4
Pre-pregnancy weight, kg	68.3 ± 13.0	68.1 ± 13.7	68.2 ± 13.4
Pre-pregnancy BMI, kg/m^2^	24.4 ± 4.3	24.3 ± 4.6	24.4 ± 4.5
**Pre-pregnancy BMI category, *n* (%)**			
BMI 18.5–24.9 kg/m^2^	645/1003 (64.3%)	652/995 (65.5%)	1297/1998 (64.9%)
BMI 25.0–29.9 kg/m^2^	243/1003 (24.2%)	218/995 (21.9%)	461/1998 (23.1%)
BMI 30.0–40.0 kg/m^2^	115/1003 (11.5%)	125/995 (12.6%)	240/1998 (12.0%)
**GWG, kg**	13.9 ± 5.3	13.9 ± 5.3	13.9 ± 5.3
**GWG classified according to the IOM, *n* (%) ^b^**			
Inadequate GWG	223/997 (22.4%)	201/988 (20.3%)	424/1985 (21.4%)
Adequate GWG	334/997 (33.5%)	344/988 (34.8%)	678/1985 (34.2%)
Excessive GWG	440/997 (44.1%)	443/988 (44.8%)	883/1985 (44.5%)
**GDM, *n* (%)**	98/984 (10.0%)	104/939 (11.1%)	202/1923 (10.5%)
**Educational level**			
General secondary school	151/1002 (15.1%)	164/992 (16.5%)	315/1994 (15.8%)
Intermediate secondary school	435/1002 (43.4%)	413/992 (41.6%)	848/1994 (42.5%)
High school	416/1002 (41.5%)	415/992 (41.8%)	831/1994 (41.7%)
**Country of birth, *n* (%)**			
Germany	889/1003 (88.6%)	886/992 (89.0%)	1775/1995 (88.8%)
Others	114/1003 (11.4%)	106/992 (10.7%)	220/1995 (11.0%)
**Primiparous, *n* (%)**	620/1003 (61.8%)	529/995 (53.2%)	1149/1998 (57.5%)
**Smoking in late pregnancy, *n* (%)**	41/952 (4.3%)	50/938 (5.3%)	91/1890 (4.8%)

Abbreviations: GDM: gestational diabetes mellitus; GWG: gestational weight gain; IOM: Institute of Medicine; BMI: body mass index. ^a^ mean ± SD (all such values). ^b^ GWG as classified by the IOM [24].

**Table 2:** Weight outcomes in intervention and control groups.

	Intervention	Control	Effect Size (95% CI)	*p*	Adjusted Effect Size (95% CI)	Adjusted *p*
**Maternal weight characteristics**						
First measured weight, kg ^a,b^	69.8 ± 13.1	68.9 ± 13.9				
Weight T1_pp_, kg ^b^	73.8 ± 13.4	73.0 ± 13.7	0.62 (−1.10, 2.35)	0.479	0.53 (−0.32, 1.39) ^d^	0.221 ^d^
Weight T2_pp_, kg ^c^	69.7 ± 13.7	69.8 ± 14.4	−0.26 (−2.10, 1.58)	0.783	−0.04 (−0.97, 0.89) ^d^	0.935 ^d^
**Short-term PPWR—T1_pp_^b^**						
WL since delivery, kg ^e^	9.9 ± 3.4	9.7 ± 3.4	0.20 (−0.15, 0.56)	0.267	0.11 (−0.22, 0.44) ^f^	0.500 ^f^
PPWR, kg	4.0 ± 4.8	4.3 ± 4.8	−0.12 (−0.94, 0.71)	0.783	−0.16 (−0.95, 0.63) ^d^	0.694 ^d^
					−0.14 (−0.96, 0.68) ^g^	0.742 ^g^
PPWR > 5 kg, *n* (%)	372/973 (38.2%)	382/934 (40.9%)	0.95 (0.71, 1.28)	0.728	0.94 (0.71, 1.24) ^d^	0.643 ^d^
Women ≤ pre-pregnancy weight, *n* (%)	110/973 (11.3%)	136/934 (14.6%)	0.75 (0.56, 1.01)	0.055	0.74 (0.56, 0.98) ^h^	0.037 ^h^
**Long-term PPWR—T2_pp_^c^**						
WL since delivery, kg ^e^	14.3 ± 5.9	13.4 ± 6.0	0.86 (0.21, 1.51)	0.009	0.85 (0.22, 1.49) ^f^	0.008 ^f^
PPWR, kg	−0.2 ± 4.8	0.6 ± 5.2	−0.63 (−1.44, 0.19)	0.132	−0.69 (−1.57, 0.19) ^d^	0.123 ^d^
					−0.74 (−1.55, 0.07) ^g^	0.075 ^g^
PPWR > 5 kg, *n* (%)	96/843 (11.4%)	123/832 (14.8%)	0.81 (0.55, 1.19)	0.277	0.72 (0.47, 1.11) ^d^	0.142 ^d^
Women ≤ pre-pregnancy weight, *n* (%)	341/843 (40.5%)	334/832 (40.1%)	1.02 (0.84, 1.23)	0.876	1.02 (0.84, 1.25) ^h^	0.823 ^h^

Abbreviations: GEE: generalized estimating equations; T1_pp_: 6–8 weeks pp; T2_pp_: 12th month pp; *p*: *p* value; pp: postpartum; PPWR: pp weight retention; WL: weight loss. ^a^ Mean ± SD (all such values). ^b^ Included are all women with available weight data for T1_pp_ (IV: *n* = 973; C: *n* = 934). ^c^ Excluded are pregnant women (*n* = 75) and women lost to follow-up (*n* = 215). ^d^ Linear or binary logistic regression models fit using GEEs adjusted for pre-pregnancy BMI, pre-pregnancy age, parity, gestational age at inclusion, time of the pp weight assessment (T1_pp_ or T2_pp_). ^e^ Included are all women with available weight data at delivery and pp (T1_pp_: IV: *n* = 970; C: *n* = 929; T2_pp_: IV: *n* = 841; C: *n* = 828). ^f^ Linear or binary logistic regression models fit using GEEs adjusted for pre-pregnancy BMI, pre-pregnancy age, parity, time of the weight assessment (gestational week at birth and T1_pp_ or T2_pp_). ^g^ Post-hoc adjustment for exclusive breastfeeding. ^h^ Linear or binary logistic regression models fit using GEEs adjusted for pre-pregnancy BMI, pre-pregnancy age, parity, time of the pp weight assessment (T1_pp_ or T2_pp_).

**Table 3:** Breastfeeding behavior of women in intervention and control groups.

	Intervention *n* (%)	Control *n* (%)	Effect Size (95% CI)	*p*	Adjusted Effect Size ^b^ (95% CI)	Adjusted *p* ^b^
**Breastfeeding behavior**						
**Any breastfeeding**						
No	127/828 (15.3%)	119/804 (14.8%)				
Yes	701/828 (84.7%)	685/804 (85.2%)	0.95 (0.50, 1.81)	0.887	0.98 (0.55, 1.76)	0.954
Breastfeeding duration, months ^a,c^	6.6 ± 3.3	6.4 ± 3.2	0.23 (−0.15, 0.60)	0.236	0.23 (−0.07, 0.54)	0.135
Breastfeeding ≥ 12 months	216/701 (30.8%)	183/685 (26.7%)	1.22 (0.82, 1.83)	0.331	1.33 (0.89, 1.99)	0.170
**Exclusive breastfeeding**						
No	85/673 (12.6)	103/661 (15.6)				
Yes	588/673 (87.4%)	558/661 (84.4%)	1.32 (1.05, 1.67)	0.019	1.49 (1.22, 1.81)	<0.001
Duration of exclusive breastfeeding, months ^a^	4.8 ± 1.8	4.7 ± 1.7	0.09 (−0.05, 0.24)	0.216	0.15 (−0.02, 0.32)	0.075
Meeting breastfeeding recommendations ^d^	476/587 (81.1%)	446/553 (80.7%)	1.08 (0.95, 1.23)	0.232	1.08 (0.94, 1.24)	0.267

Abbreviations: *p*: *p* value; T2_pp_: 12th month postpartum. ^a^ Mean ± SD (all such values). ^b^ Adjusted for maternal pre-pregnancy age, pre-pregnancy BMI, parity, T2_pp_. ^c^ Without participants that still breastfeed. ^d^ Included are participants that exclusively breastfed. Breastfeeding recommendations are defined according to [32].

**Table 4:** Predictors of long-term PPWR.

PPWR at T2_pp_	Effect Size (95% CI)	*p*	Adjusted Effect Size (95% CI)	Adjusted *p*
**Pre-pregnancy BMI category ^a^**		0.021		0.012
BMI 18.5–24.9 kg/m^2^	Reference		Reference	
BMI 25.0–29.9 kg/m^2^	0.47 (−0.10, 1.05)	0.106	1.01 (0.28, 1.74)	0.006
BMI 30.0–40.0 kg/m^2^	−0.72 (−1.48, 0.03)	0.060	0.44 (−0.82, 1.70)	0.494
**Age categories (pre-pregnancy) ^b^**		0.002		0.013
18–25 years	Reference		Reference	
26–35 years	−1.29 (−2.04, −0.55)	0.001	−1.13 (−1.89, −0.38)	0.003
36–43 years	−1.39 (−2.34, −0.44)	0.004	−1.00 (−1.98, −0.02)	0.045
**Educational level ^c^**		0.001		0.001
General secondary school	Reference		Reference	
Intermediate secondary school	−0.45 (−1.18, 0.27)	0.221	−0.57 (−1.29, 0.16)	0.129
High school	−1.21 (−1.93, −0.48)	0.001	−1.28 (−2.02, −0.54)	0.001
**Excessive GWG according to the IOM ^c,d^**		<0.001		<0.001
Non-excessive GWG	Reference		Reference	
Excessive GWG	2.04 (1.56, 2.53)	<0.001	2.25 (1.74, 2.77)	<0.001
**Parity ^e^**		0.002		0.002
Primiparous	Reference		Reference	
Multiparous	0.77 (0.29, 1.25)	0.002	0.77 (0.27, 1.27)	0.002
**PPWR at T1_pp_^c^**		<0.001		<0.001
PPWR ≤ 5 kg	Reference		Reference	
PPWR > 5 kg	3.84 (3.38, 4.30)	<0.001	3.82 (3.36, 4.28)	<0.001
**Any breastfeeding ^c^**		0.295		0.280
No	Reference		Reference	
Yes	0.37 (−0.32, 1.05)	0.295	0.38 (−0.31, 1.06)	0.280
**Exclusive breastfeeding ^c^**		0.002		0.005
No	Reference		Reference	
Yes	−1.24 (−2.02, −0.46)	0.002	−1.13 (−1.92, −0.33)	0.005
**Antenatal well-being ^c^**		0.447		0.500
Moderate to high	Reference		Reference	
Low	0.20 (−0.31, 0.71)	0.447	0.18 (−0.33, 0.69)	0.500
**PPD ^c^**		0.261		0.268
No	Reference		Reference	
Yes	0.51 (−0.38, 1.40)	0.261	0.50 (−0.38, 1.38)	0.268

Depicted is the regression coefficient of the PPWR in kg at T2_pp_ along with the 95% confidence interval (CI). Abbreviations: GWG: Gestational weight gain; IOM: Institute of Medicine; *p*: *p* value; pp: postpartum; PPD: pp depression; PPWR: pp weight retention; T1_pp_: 6–8 weeks pp; T2_pp_: 12th month pp. ^a^ Linear regression model adjusted for pre-pregnancy age, parity, group assignment, gestational age at inclusion, T2_pp_. ^b^ Linear regression model adjusted for pre-pregnancy BMI, parity, group assignment, gestational age at inclusion, T2_pp_. ^c^ Linear regression model adjusted for pre-pregnancy BMI, pre-pregnancy age, parity, group assignment, gestational age at inclusion, T2_pp_. ^d^ Excessive GWG as defined by the IOM [24]. ^e^ Linear regression model adjusted for pre-pregnancy BMI, pre-pregnancy age, group assignment, gestational age at inclusion, T2_pp_.

**Table 5:** Predictors of exclusive breastfeeding.

	Exclusive Breastfeeding		No Exclusive Breastfeeding		Effect Size (95% CI)	*p*	Adjusted Effect Size (95% CI)	Adjusted *p*
	*n*	%	*n*	%				
**Pre-pregnancy BMI category ^a^**						0.001	0.001	0.001
BMI 18.5–24.9 kg/m^2^	781/885	88.2	104/885	11.8	Reference		Reference	
BMI 25.0–29.9 kg/m^2^	253/306	82.7	53/306	17.3	0.64 (0.44, 0.91)	0.014	0.60 (0.41, 0.87)	0.006
BMI 30.0–40.0 kg/m^2^	112/143	78.3	31/143	21.7	0.48 (0.31, 0.75)	0.001	0.48 (0.30, 0.76)	0.002
**Age categories (pre-pregnancy) ^b^**						0.725		0.124
18–25 years	131/151	86.8	20/151	13.2	Reference		Reference	
26–35 years	873/1013	86.2	140/1013	13.8	0.95 (0.58, 1.58)	0.848	0.81 (0.49, 1.36)	0.430
36–43 years	142/169	84	27/169	16.0	0.80 (0.43, 1.50)	0.491	0.53 (0.28, 1.02)	0.057
**Educational level ^c^**						0.022		0.024
General secondary school	113/144	78.5	31/144	21.5	Reference		Reference	
Intermediate secondary school	474/549	86.3	75/549	13.7	1.73 (1.09, 2.76)	0.021	1.80 (1.11, 2.92)	0.017
High school	559/640	87.3	81/640	12.7	1.89 (1.19, 3.00)	0.007	1.93 (1.19, 3.13)	0.008
**Excessive GWG according to the IOM ^c,d^**						0.033		0.519
Non-excessive GWG	612/692	88.4	80/692	11.6	Reference		Reference	
Excessive GWG	478/567	84.3	89/567	15.7	0.70 (0.51, 0.97)	0.033	0.89 (0.62, 1.27)	0.519
**Antenatal well-being ^c^**						0.337		0.350
Moderate to high	742/855	86.8	113/855	13.2	Reference		Reference	
Low	369/435	84.8	66/435	15.2	0.85 (0.61, 1.18)	0.337	0.85 (0.61, 1.19)	0.350
**PPD ^c^**						0.039		0.105
No	1005/1164	86.3	159/1164	13.7	Reference		Reference	
Yes	73/93	78.5	20/93	21.5	0.58 (0.34, 0.97)	0.039	0.64 (0.38, 1.10)	0.105

Abbreviations: GWG: Gestational weight gain; IOM: Institute of Medicine; *p*: *p* value; pp: postpartum; PPD: pp depression; T2_pp_: 12th month pp. ^a^ Binary logistic regression model adjusted for pre-pregnancy age, parity, group assignment, T2_pp_. ^b^ Binary logistic regression model adjusted for pre-pregnancy BMI, parity, group assignment, T2_pp_. ^c^ Binary logistic regression model adjusted for pre-pregnancy age, pre-pregnancy BMI, parity, group assignment, T2_pp_. ^d^ Excessive GWG as defined by the IOM [24].

## Supplementary Materials

The following are available online at https://www.mdpi.com/2077-0383/8/6/876/s1, Table S1: Characteristics of women entering the follow-up compared to women lost to follow-up, Table S2: Sensitivity analyses on short-term and long-term PPWR, Table S3: Factors influencing the decision to breastfeed (any breastfeeding).

## Abbreviations

BMI	Body mass index
C	Control group
CI	Confidence interval
EPDS	Edinburgh Postnatal Depression Scale
GDM	Gestational diabetes mellitus
GEE	Generalized estimating equation
GeliS	“Gesund leben in der Schwangerschaft”/ “healthy living in pregnancy”
GWG	Gestational weight gain
IOM	Institute of Medicine
IV	Intervention group
KiGGS	German Health Interview and Examination Survey for Children and Adolescents
OR	Odds ratio
PP	Postpartum
PPD	Postpartum depression
PPWR	Postpartum weight retention
QT2_pp_	Set of questionnaires provided at T2_pp_
RCT	Randomized-controlled trial
SD	Standard deviation
T1_pp_	Time point of first data collection postpartum
T2_pp_	Time point of second data collection postpartum
V0	Visit 0, time of recruitment (before the 12th week of gestation)
WHO	World Health Organization
WHO-5	5-item World Health Organization Well-Being Index
WL	Weight loss
