# Supplementary material for: Effects of a Lifestyle Intervention in Routine Care on Short- and Long-Term Maternal Weight Retention and Breastfeeding Behavior—12 Months Follow-up of the Cluster-Randomized GeliS Trial
Source: J Clin Med. 2019 Jun 19;8(6):876. doi: 10.3390/jcm8060876 (PMC6616390; doi:10.3390/jcm8060876)
Supplement: Supplementary file 1 [file jcm-08-00876-s001.pdf]

Table S1: Characteristics of women entering the follow-up compared to women lost to follow-up.

	Participants in 12 months follow-up			Lost to follow-up		
	**Intervention (*n* = 902)**	**Control (*n* = 881)**	**Total (*n* = 1783)**	**Intervention (*n* = 101)**	**Control (*n* = 114)**	**Total (*n* = 215)**
**Maternal characteristics**						
**Pre-pregnancy age, years**[a]	30.4 ± 4.2	30.6 ± 4.5	30.5 ± 4.4	29.0 ± 4.6	29.0 ± 5.0	29.0 ± 4.8
**Pre-pregnancy weight, kg**	68.6 ± 13.1	68.0 ± 13.5	68.3 ± 13.3	66.0 ± 11.5	68.1 ± 15.0	67.1 ± 13.5
**First measured weight, kg**	70.1 ± 13.2	68.9 ± 13.8	69.5 ± 13.5	67.4 ± 11.6	69.1 ± 15.0	68.3 ± 13.5
**Pre-pregnancy BMI, kg/m^2**	24.4 ± 4.3	24.3 ± 4.6	24.4 ± 4.5	24.0 ± 3.9	24.5 ± 4.7	24.2 ± 4.4
**Pre-pregnancy BMI category, n (%)**						
BMI 18.5–24.9 kg/m^2	578/902 (64.1%)	577/881 (65.5%)	1155/1783 (64.8%)	67/101 (66.3%)	75/114 (65.8%)	142/215 (66.0%)
BMI 25.0–29.9 kg/m^2	217/902 (24.1%)	194/881 (22.0%)	411/1783 (23.1%)	26/101 (25.7%)	24/114 (21.1%)	50/215 (23.3%)
BMI 30.0–40.0 kg/m^2	107/902 (11.9%)	110/881 (12.5%)	217/1783 (12.2%)	8/101 (7.9%)	15/114 (13.2%)	23/215 (10.7%)
**GWG, kg**	14.0 ± 5.2	14.0 ± 5.2	14.0 ± 5.2	13.4 ± 5.5	13.1 ± 5.7	13.2 ± 5.6
**GDM, n (%)**	89/887 (10.0%)	82/832 (9.9%)	171/1719 (9.9%)	9/97 (9.3%)	22/107 (20.6%)	31/204 (15.2%)
**Educational level**						
General secondary school	120/901 (13.3%)	140/880 (15.9%)	260/1781 (14.6%)	31/101 (30.7%)	24/112 (21.4%)	55/213 (25.8%)
Intermediate secondary school	392/901 (43.5%)	363/880 (41.3%)	755/1781 (42.4%)	43/101 (42.6%)	50/112 (44.6%)	93/213 (43.7%)
High school	389/901 (43.2%)	377/880 (42.8%)	766/19781 (43.0%)	27/101 (26.7%)	38/112 (33.9%)	65/213 (30.5%)
**Country of birth, n (%)**						
Germany	801/902 (88.8%)	798/880 (90.7%)	1599/1782 (89.7%)	88/101 (87.1%)	88/112 (78.6%)	176/213 (82.6%)
Others	101/902 (11.2%)	82/880 (9.3%)	183/1782 (10.3%)	13/101 (12.9%)	24/112 (21.4%)	37/213 (17.4%)
**Primiparous, n (%)**	568/902 (63.0%)	477/881 (54.1%)	1045/1783 (58.6%)	52/101 (51.5%)	52/114 (45.6%)	104/215 (48.4%)
**Smoking in late pregnancy, n (%)**	35/864 (4.1%)	37/845 (4.4%)	72/1709 (4.2%)	6/88 (6.8%)	13/93 (14.0%)	19/181 (10.5%)

Abbreviations: GDM: Gestational diabetes mellitus; GWG: Gestational weight gain.

[a] mean ± SD (all such values)

Table S2: Sensitivity analyses on short-term and long-term PPWR.

	Intervention	Control	Effect size (95% CI)	p	Adjusted effect size[d] (95% CI)[f]	Adjusted p[f]
**Short-term PPWR–T1$_{pp}$**						
**PPWR, kg, model 1[b]**	4.0 ± 4.8[a]	4.3 ± 4.8	-0.12 (-0.94, 0.71)	0.783	-0.16 (-0.95, 0.63)	0.694
**PPWR, kg, model 2[c]**	4.0 ± 4.8	4.3 ± 4.8	-0.12 (-0.94, 0.71)	0.782	-0.18 (-0.97, 0.61)	0.662
**Long-term PPWR–T2$_{pp}$**						
**PPWR, kg, model 1[d]**	-0.2 ± 4.8	0.6 ± 5.2	-0.63 (-1.44, 0.19)	0.132	-0.69 (-1.57, 0.19)	0.123
**PPWR, kg, model 2[e]**	-0.2 ± 4.8	0.6 ± 5.2	-0.61 (-1.44, 0.21)	0.146	-0.68 (-1.57, 0.21)	0.133

Abbreviations: GEE: generalized estimating equations; T1$_{pp}$: 6-8 weeks pp; T2$_{pp}$: 12th month pp; p: p value; pp: postpartum; PPWR: pp weight retention; V0: Visit 0 and time of recruitment.

[a] Mean ± SD (all such values).

[b] Included are all women with available weight data for T1$_{pp}$. Self-reported pre-pregnancy weight was used if the measured weight at V0 was missing (IV: *n* = 973; C: *n* = 934).

[c] Included are all women with available measured weight data for T1$_{pp}$ (IV: *n* = 964; C: *n* = 917).

[d] Excluded are pregnant women and women lost to follow-up. Self-reported pre-pregnancy weight was used if the measured weight at V0 was missing (IV: *n* = 843; C: *n* = 832).

[e] Excluded are pregnant women, women lost to follow-up and women with missing measured weight data at V0 (IV: *n* = 839; C: *n* = 817).

[f] Linear regression models fit using GEEs adjusted for pre-pregnancy BMI, pre-pregnancy age, parity, gestational age at inclusion, time of the pp weight assessment (T1$_{pp}$ or T2$_{pp}$).

Table S3: Factors influencing the decision to breastfeed (any breastfeeding).

	Any Breastfeeding		No breastfeeding					
	n	%	*n*	%	Effect size (95% CI)	p	Adjusted effect size (95% CI)	Adjusted p
**Pre-pregnancy BMI category**[a]						**0.002**		**0.002**
BMI 18.5–24.9 kg/m^2	924/1066	86.7	142/1066	13.3	Reference		Reference	
BMI 25.0–29.9 kg/m^2	313/372	84.1	59/372	15.9	0.82 (0.59, 1.13)	0.225	0.83 (0.59, 1.15)	0.259
BMI 30.0–40.0 kg/m^2	149/194	76.8	45/194	23.2	0.51 (0.35, 0.74)	< 0.001	0.51 (0.35, 0.74)	< 0.001
**Age categories (pre-pregnancy)**[b]						**0.337**		**0.259**
18–25 years	158/194	81.4	36/194	18.6	Reference		Reference	
26–35 years	1051/1229	85.5	178/1229	14.5	1.35 (0.91, 2.00)	0.141	1.40 (0.93, 2.09)	0.105
36–43 years	176/208	84.6	32/208	15.4	1.25 (0.74, 2.11)	0.397	1.42 (0.82, 2.45)	0.210
**Educational level**[c]						**< 0.001**		**< 0.001**
General secondary school	154/215	71.6	61/215	28.4	Reference		Reference	
Intermediate secondary school	567/693	81.8	126/693	18.2	1.78 (1.25, 2.54)	0.001	1.71 (1.19, 2.45)	0.004
High school	664/723	91.8	59/723	8.2	4.46 (2.99, 6.64)	< 0.001	4.08 (2.70, 6.15)	< 0.001
**Excessive GWG according to the IOM**[c, d]						**0.273**		**0.690**
Non-excessive GWG	720/832	86.5	112/832	13.5	Reference		Reference	
Excessive GWG	586/693	84.6	107/693	15.4	0.85 (0.64, 1.14)	0.273	0.94 (0.69, 1.28)	0.690
**Antenatal well-being**[c]						**0.007**		**0.018**
Moderate to high	882/1015	86.9	133/1015	13.1	Reference		Reference	
Low	459/561	81.8	102/561	18.2	0.68 (0.51, 0.90)	0.007	0.71 (0.53, 0.94)	0.018
**PPD**[c]						**< 0.001**		**< 0.001**
No	1208/1401	86.2	193/1401	13.8	Reference		Reference	
Yes	97/133	72.9	36/133	27.1	0.43 (0.29, 0.65)	< 0.001	0.45 (0.29, 0.68)	< 0.001

Abbreviations: GWG: Gestational weight gain; IOM: Institute of Medicine; p: p value; pp: postpartum; PPD: pp depression; $T2_{pp}$: 12th month pp.

[a] Binary logistic regression model adjusted for pre-pregnancy age, parity, group assignment,$T2_{pp}$.

[b] Binary logistic regression model adjusted for pre-pregnancy BMI, parity, group assignment, $T2_{pp.}$

[c] Binary logistic regression model adjusted for pre-pregnancy age, pre-pregnancy BMI, parity, group assignment, $T2_{pp}$.

[d] Excessive GWG as defined by the IOM [24].
